# R^2^GDN: RepGhost based residual dense network for image super-resolution

**DOI:** 10.1371/journal.pone.0338432

**Published:** 2025-12-12

**Authors:** Tianyu Li, Xiaoshi Jin, Qiang Liu, Xi Liu, Zehang Yuan, Tianyang Liang, Jia Lou, Yangfan Rao

**Affiliations:** 1 School of Information Science and Engineering, Shenyang University of Technology, Shenyang, China; 2 School of Information Science and Engineering, Shenyang University of Technology, Shenyang, China; 3 Engineering Training Center, Shenyang University of Technology, Shenyang, China; 4 School of Information Science and Engineering, Shenyang University of Technology, Shenyang, China; 5 School of Artificial Intelligence, Shenyang University of Technology, Shenyang, China; 6 School of Artificial Intelligence, Shenyang University of Technology, Shenyang, China; 7 School of Artificial Intelligence, Shenyang University of Technology, Shenyang, China; 8 School of Information Science and Engineering, Shenyang University of Technology, Shenyang, China; Institut Sains dan Teknologi Terpadu Surabaya, INDONESIA

## Abstract

This study introduces a novel lightweight image super-resolution reconstruction network aimed at mitigating the challenges associated with computational complexity and memory consumption in existing super-resolution reconstruction networks. The proposed network optimizes its architecture through feature reuse and structural reparameterization, rendering it more suitable for deployment in edge computing environments. Specifically, we have developed a new lightweight reparameterization layer that derives redundant features from intrinsic features using low-cost operations and integrates them with reparameterization techniques to enhance efficient feature utilization. Furthermore, an efficient deep feature extraction module named RGAB has been designed, which retains dense connections, local feature integration, and local residual learning mechanisms while incorporating addition operations for feature integration. The resultant network, termed R^2^GDN, exhibits a significant reduction in model parameters and improved inference speed. Compared to performance-oriented super-resolution algorithms, our model reduces the number of parameters by approximately 95% and enhances inference speed by 86.8% on the edge device. When benchmarked against lightweight super-resolution algorithms, our model maintains a lower parameter count and achieves a 0.74% improvement in the structural similarity index (SSIM) on the BSD100 dataset for 4 × super-resolution reconstruction. Experimental results demonstrate that R^2^GDN effectively balances network performance and complexity.

## 1. Introduction

Image super-resolution reconstruction is a classic and challenging low-level vision task in computer vision, which is an ill-posed problem where obtaining a unique high-resolution image from a low-resolution input is difficult. The concept of super-resolution was first introduced by Harris [1], and has since gained increasing attention and become a hot research topic in image processing. The development of efficient computing hardware and complex algorithms [2] has unlocked the great potential of deep learning in handling unstructured data. This, in turn, has led to the rapid advancement of deep-learning-based image super-resolution methods. SRCNN [3] (Super-Resolution Convolutional Neural Network) was the first to apply convolutional neural networks to this task.

Research on deep learning theory [4] has shown that the solution space of deep neural networks can be expanded by deepening and widening the network structure. Many network designs focus on increasing depth and width for better performance. However, as networks get deeper, training VGG – based networks becomes harder, leading to the development of ResNet with skip connections [5–6]. EDSR [7] by Lim et al. removed the BN layer for better performance in image super-resolution. EDSR (Enhanced Deep Super-Resolution) not only increases network depth but also boosts feature dimensions. RDN [8] (Residual Dense Network) by Zhang et al. uses residual dense blocks with densely connected convolutional layers to extract rich local features. SwinIR (Swin Transformer for Image Restoration) [9] leverages the hierarchical structure and sliding window mechanism of Swin Transformer for effective feature extraction and reconstruction. HAT (Hybrid Attention Transformer) [10] combines channel attention and self-attention mechanisms to activate more input pixels for reconstruction.

With technological progress, high resolution images from super resolution reconstruction contain richer details and are now widely used in medical imaging [11], remote sensing satellite imaging [12], industrial product inspection [13], and microscopic imaging [14–15].

In the domain of image super-resolution reconstruction, although performance-oriented algorithms [7–10] can obtain outstanding image reconstruction outcomes, they typically feature complex model architectures and large parameter sizes. These elements increase both the storage demands for model parameters and the memory usage of intermediate features, which restricts their deployment on devices with limited memory. Hence, developing lightweight models to strike a balance between image reconstruction quality and computational resource consumption is of vital importance [16].

FSRCNN (Fast Super-Resolution Convolutional Neural Networks) [17] is the first network to use deconvolution layers to reconstruct HR images from LR feature maps. EDSR-baseline [7] simplifies the network while maintaining the original model’s performance. DRCN (Deeply-Recursive Convolutional Network) [18] enhances network depth through its recursive structure, expanding the receptive field to capture global image information and effectively reducing the number of parameters. CARN (Cascading Residual Network) [19] uses a cascading structure and residual connections to improve feature propagation efficiency, making it suitable for lightweight super-resolution tasks with fast inference speed. IMDN (Information Multi-Distillation Network) [20] gradually extracts hierarchical features through an information distillation mechanism, enhancing feature extraction capabilities, particularly for complex scene super-resolution reconstruction. RepRFN (Reparameterized Residual Feature Network) [21] designs a multi-scale feature fusion structure, enabling the network to learn and fuse features of various scales and high-frequency edges. SwinIR-light [9], a lightweight version of SwinIR, inherits the advantages of the Swin Transformer while reducing model size and computational requirements, making it suitable for mobile devices.

Despite the lightweight achievements of these networks for super-resolution models to varying degrees, several challenges persist. These include issues of gradient disappearance or explosion; the increased computational burden from information distillation processes, which also involve a large number of parameters and are unsuitable for resource-constrained devices; increased model complexity due to multi-scale structures; high demand for computational resources; and the potential for significant degradation in reconstruction performance.

In this study, we first utilized intrinsic features to generate redundant features [22] through low-cost operations and developed a reparametrized layer for feature reuse (RG-Layer) using structural reparameterization techniques [23–24]. We then designed an efficient deep feature extraction module (RGAB, RepGhost and addition based residual dense blocks) that retains dense connections, local feature integration, and local residual learning, using addition operations for feature fusion. Finally, we constructed R^2^GDN, an efficient image super-resolution reconstruction network. Compared with performance-oriented image super-resolution reconstruction algorithms (e.g., RDN), our algorithm reduces the number of parameters by 96% and shortens the inference time on the device by 86.83%. When compared with typical lightweight super-resolution algorithms, our algorithm shows improvements in the structural similarity index (SSIM) during benchmark testing.

The method proposed in this paper does not simply assemble existing structures. Compared to traditional dense connection blocks that utilize front-end features through convolution and Concat operations, the RG-Layer designed in this paper transfers the process of fusing feature spaces, which involves deriving redundant features from essential features through inexpensive operations, to the weight space. During inference, the parallel structure can be merged into a single and efficient convolutional layer, thereby achieving rich feature representation at the minimal operational cost. The RGAB module combines the RG-Layer (which efficiently generates diverse features) with additive operations (refining features within a fixed dimension) to create an efficient local learning environment: each RG-Layer fine-tunes and enhances features based on the fusion of previous layers, rather than simply increasing the number of features. This encourages the network to learn more representative and informative features, avoiding the unbounded expansion of feature maps. The additive operation provides a more direct path for gradient propagation, in line with the idea of residual learning, which helps stabilize the training of deep networks. The effectiveness of R²GDN stems from our profound understanding of “feature redundancy” and “feature fusion,” as well as the collaborative innovation of structural reparameterization and additive dense connections, which realizes a super-resolution reconstruction model that is “wide during training and narrow during inference.”

In this paper, our main work is as follows:

1) We combine feature reuse and structural reparameterization techniques in image super-resolution reconstruction to develop a reparametrized layer for feature reuse (RG-Layer). This approach effectively reduces the number of model parameters and computational complexity through feature fusion.
2) We propose an efficient deep feature extraction module (RGAB) for fast and accurate image super-resolution reconstruction. This module achieves competitive results with a lower number of parameters, enhancing feature extraction and utilization efficiency.
3) We explore the effect of the addition operation in image super-resolution reconstruction through experiments. The addition operation, which combines features additively, enables the extraction of local dense features in images and can serve as a reference for lightweight network design. Our model achieves an excellent balance between visual quality and inference speed.

## 2. Methods

### 2.1. Structural reparameterization and feature reuse

**Fig 1:**
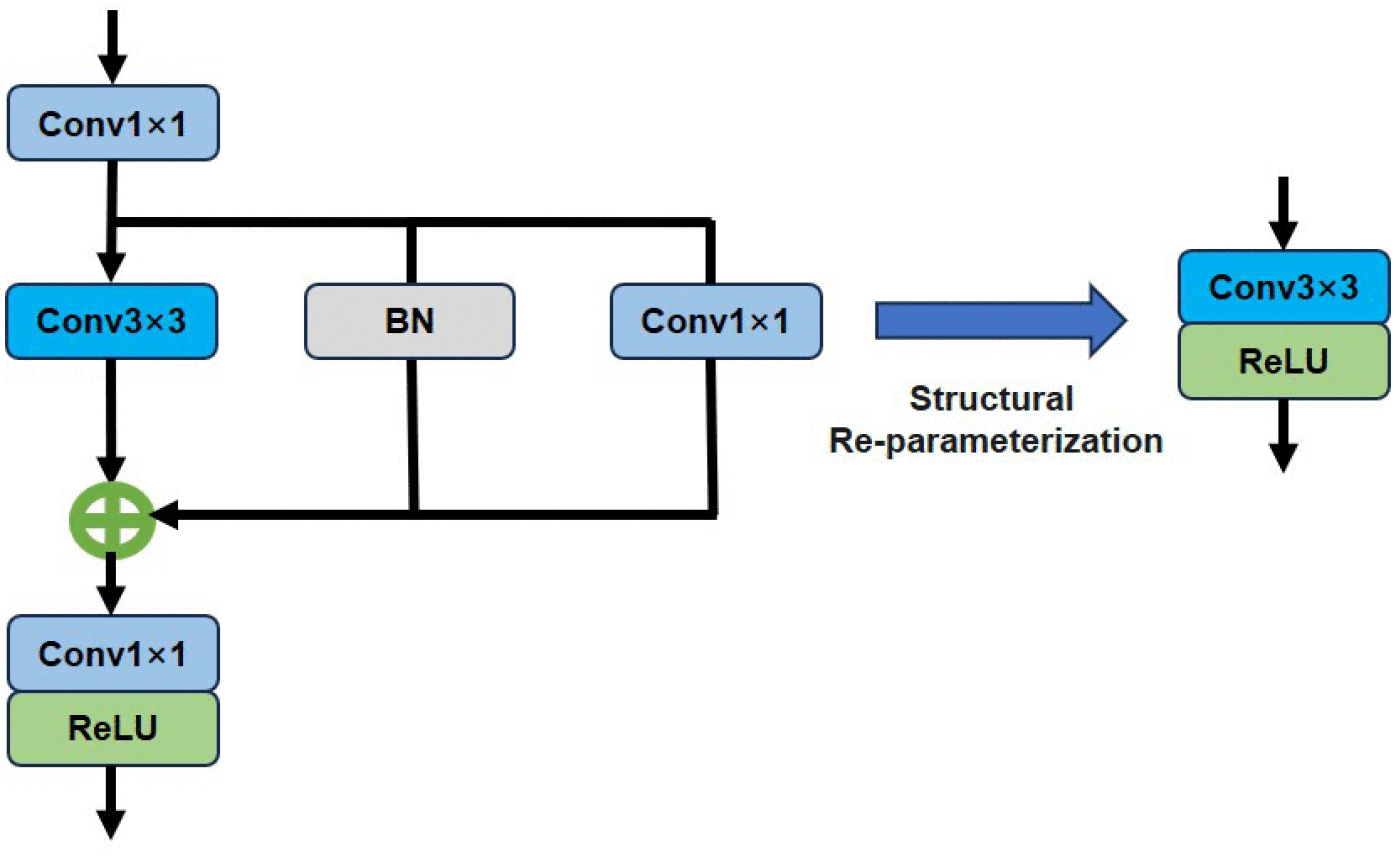
A schematic diagram of structural reparameterization using a parallel structure.

Structural reparameterization, a deep learning optimization technique, enhances model performance, efficiency, and generalization [25] by adjusting network structure to reduce parameters and computational cost. Chen et al. [24] indicate that Structural reparameterization involves multiple linear operators during training to generate diverse feature maps. During the inference process, these operators are merged into a single operator via parameter fusion for fast inference. The process of structural reparameterization is schematically illustrated in Fig 1.

**Fig 2:**
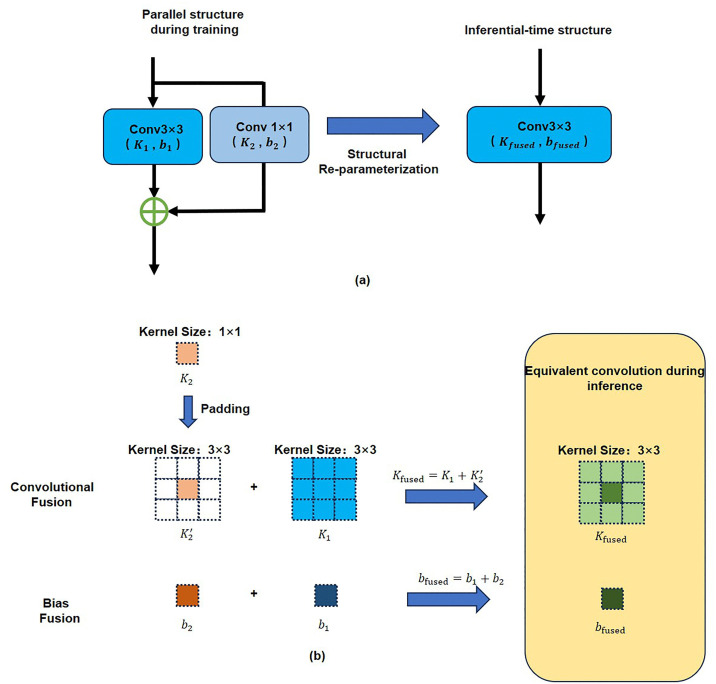
Schematic diagram of parallel structure fusion process during reasoning process. (Fig 2 (a) is a schematic diagram of the parallel structure in the network; Fig 2. (b) illustrates the fusion process of parallel structures in network inference.).

The specific reparameterization process is shown in Fig 2. This process involves not only the fusion of convolutional kernels but also the merging of bias terms. Taking a parallel structure containing a 3 × 3 convolution and a 1 × 1 convolution as an example, Fig 2(a) illustrates the macroscopic structural changes during training and inference. The core parameter fusion process is shown in Fig 2(b): the 3x3 convolutional kernel is defined as$\;K_{\text{1}}$, and the kernel $K_{\text{2}}$ of the 1 × 1 convolution is expanded to a 3 × 3 size through zero-padding to obtain $K_{\text{2}}^{\prime }$. Subsequently, the equivalent convolutional kernels $K_{\text{1}}$ and $K_{\text{2}}^{\prime }$ of all parallel branches are added element-wise to obtain the fused convolutional kernel $K_{fused}$; the equivalent biases $b_{\text{1}}$ and $b_{\text{2}}$ of all parallel branches are directly added to obtain the fused bias $b_{fused}$. The original entire parallel structure is replaced by a single convolutional layer with the fused weight $K_{fused}$ and bias $b_{fused}$. During the training phase, the model retains the complex parallel structure to capture rich image features and ensure high performance. During inference, the aforementioned reparameterization operation is applied to all eligible branches in the network, thereby transforming the entire network into a functionally equivalent but much faster and more memory-efficient architecture.

Feature reuse is a key strategy for improving the efficiency and performance of deep learning models. By reusing already computed feature maps, it avoids redundant calculations and reduces the computational load of the model. With multiple layers or operations able to share the same feature representations, feature reuse can decrease model parameters and prompt the model to learn richer and more robust feature representations. Also, it can speed up data processing and improve training efficiency. Common feature reuse methods include residual connections [6,8], dense connections [8,26], feature pyramids [27], and multi-scale feature fusion [7] and so on.

As the performance of neural network models improves, the scale of the network and the number of features increase. The issue of feature redundancy has gradually received attention from researchers [22]. In the task of image super-resolution reconstruction, there are also large numbers of similar features [28]. Obtaining these similar features repeatedly through regular convolution operations leads to a significant waste of computational resources. Therefore, some scholars have proposed using regular convolution to generate some intrinsic features and then using low-cost operations (such as depth-wise convolution, shift operations, etc.) to obtain redundant features. By concatenating these two parts of features, the completeness of the output features can be guaranteed while effectively reducing the number of model parameters and computational requirements. Structural reparameterization moves the fusion process from the feature space to the weight space, and is considered an implicit feature reuse method.

In deep learning, feature fusion is commonly achieved through concatenation and addition operations. Concatenation combines two or more tensors along a specified dimension to generate a larger tensor, increasing channels or feature dimensions. This enables later layers to better capture the relationships between different features. Addition sums two tensors element-wise to generate a larger tensor, facilitating gradient flow and enhancing training stability while reducing gradient vanishing in deep networks.

**Fig 3:**
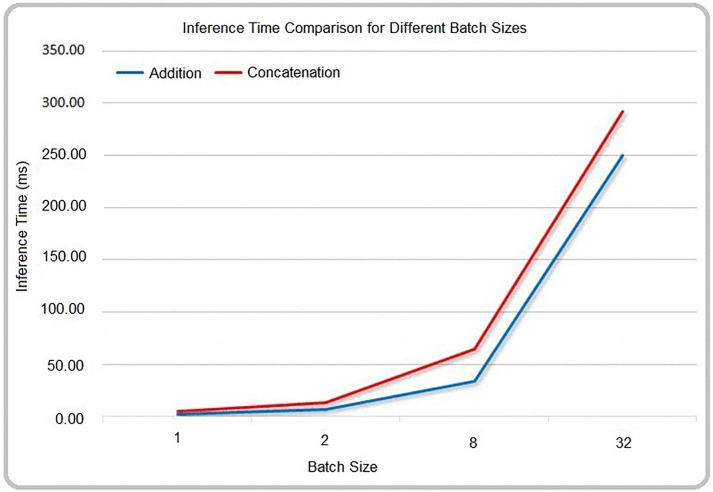
Comparison of inference times between concatenation and addition operations [24].

Although these two feature fusion methods do not introduce additional parameters or FLOPs in the network, researchers [24] have verified that, under the same batch size, the Addition operation has a lower computational cost and shorter runtime compared to the Concatenation operation, as shown in Fig 3.

### 2.2. RepGhost and addition based residual dense blocks—RGABs

**Fig 4:**
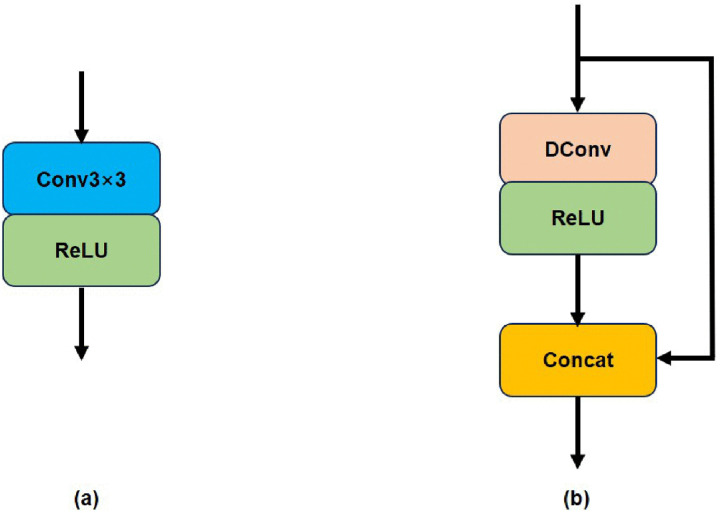
Schematic diagram of traditional convolutional structure and Ghost module structure. (Fig 4(a) is the structure of traditional convolutional; Fig 4(b) is the structure of Ghost module.).

Unlike traditional convolution-activation operations, the Ghost module [22] uses regular convolution to generate some intrinsic features and then employs low-cost operations to obtain redundant features. These two sets of features are concatenated to ensure complete output features by the concat operation while effectively reducing the number of model parameters and computational requirements, as depicted in Fig 4.

**Fig 5:**
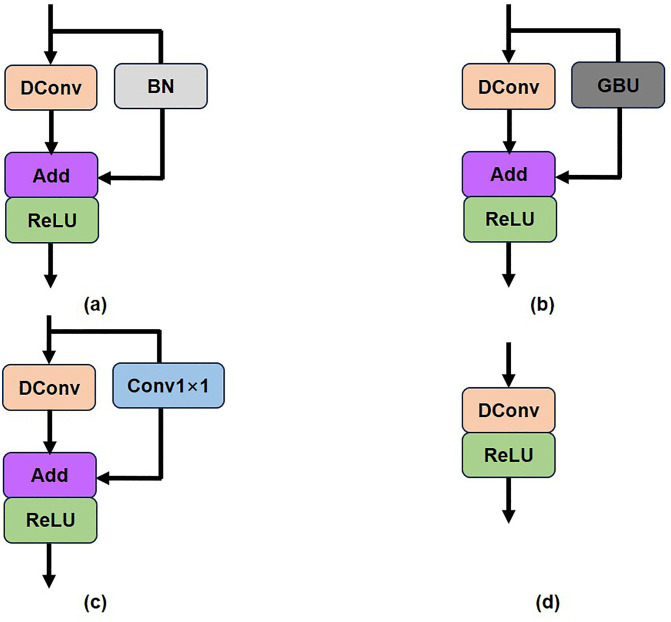
Evolution from the RepGhost module used in classification networks to the RG-Layer used in image super-resolution. (To highlight the changes in functional structure, we have omitted the 1 × 1 convolution used for channel number transformation during input and output. Fig 5 (a) shows the RepGhost module used in classification networks; Fig 5 (b) shows the RG-Layer of SR network with gated BN unit; Fig 5 (c) shows the further advancement of lightweight RG-Layer; Fig 5 (d) is the re-parameterized structure during inference.).

Based on this, we have made several adjustments and finally derived our RG-Layer, as shown in Fig 5. Fig 5(a) shows the RepGhost module [24] used in classification networks, which replaces the concat operation in Fig 4(b) with an Addition operation to enhance efficiency. To comply with structural reparameterization rules, the ReLU operation of nonlinear computation is moved after the Addition operation, and Batch Normalization (BN) is added to the identity mapping branch to introduce nonlinearity during training, making the structure more flexible.

The RG-Layer of our image super-resolution reconstruction network is depicted in Fig 5 (b) and (c). While batch normalization (BN) can ease network training and prevent overfitting, for image super-resolution reconstruction, it normalizes image color distribution, degrading the original contrast information and thus the super-resolution network’s output quality. Instead of removing the BN layer, we designed a gated structure with trainable parameters to control its effect range, mitigating BN’s negative impact in this task, as shown in Fig 5 (b).

Gated mechanisms are widely used in recurrent neural networks (RNNs) [29] to control the flow of information and improve the model’s ability to learn long-term dependencies. This mechanism dynamically adjusts the transmission of information by learning gating parameters. Inspired by these successes, recent studies have explored the application of gating mechanisms in other domains. For instance, Wang et al. [30] combined gating mechanisms with normalization methods in the context of image restoration tasks, demonstrating that such combinations can enhance feature extraction and improve overall performance.

Drawing inspiration from these advances, we designed a Gated BN Unit (GBU) to address the limitations of traditional BN in image super-resolution tasks. Specifically, we created two gating mechanisms, each consisting of a linear layer followed by an activation function (e.g., sigmoid), to output a value between 0 and 1. This value indicates the degree to which the BN layer should be activated. The process can be mathematically expressed as:


y=(1−α)·x+β·BN(x) (1)

Where α and β are trainable gating parameters that determine how much input should be retained and the extent to which the BN layer’s normalization effect should be applied.

Inspired by the literature [30] (Wang et al., combining gating with normalization for image restoration), we designed the GBU. Its form y=(1−α)·x+β·BN(x) aims to allow the network to adaptively determine the proportion of retaining the original input and utilizing BN features through learnable parameters α and β. Our original intention was to let the network adaptively find the optimal balance between the BN layer and the original identity mapping. Imposing constraints (such as making α+β=1, or restricting the value range to [0,1] through Sigmoid) is equivalent to us presetting the answer in advance, which may limit the model’s expressive power. Without constraints, the network may discover more optimal feature combination methods beyond human intuition. From the perspective of exploratory research, it is completely scientific not to impose constraints.

**Fig 6:**
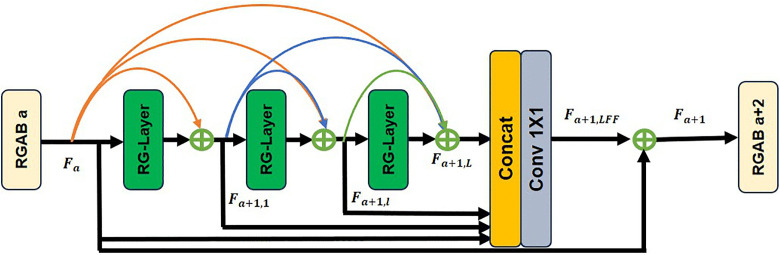
Structure of the RepGhost and addition based residual dense block (RGAB).

To further advance model lightweighting, we designed the structure in Fig 5 (c). Compared to Fig 5 (a) and 5 (b), we replaced the parallel structure with a 1 × 1 convolution branch. The 1 × 1 convolution has trainable parameters that can learn linear transformations of input features and help improve gradient flow [31] in deep networks. The structure in Fig 5 (c) can be re-parameterized to Fig 5 (d) during inference, enabling fast inference for the super-resolution network. Our RG-Layer only contains depth-wise separable convolutions (DConv) and activation functions during inference, making it more suitable for edge devices [23]. Based on the dense connection layer, local feature fusion, and local residual learning [8], we designed a lightweight RGAB (RepGhost and addition based residual dense blocks) module, as shown in Fig 6. The continuous memory mechanism is implemented by passing the features from the previous RGAB’s output to each RG-Layer of the current RGAB.

Let $F_{a}$ denote the output of the a-th RGAB module (the input of the (a+1)-th RGAB module), and $F_{a+\text{1}}$ denote the output of the (a+1)-th RGAB module, as shown in Fig 6. Both have G features. The output of the l-th RG-Layer in the i-th RGAB module can be expressed as:


$$F_{a+\text{1},l}=Act\{W_{a+\text{1},l}\left[Add\left(F_{a},F_{a+\text{1},\text{1}},\ldots \ldots ,F_{a+\text{1},l-\text{1}}\right)\right\}$$ (2)

In the formula 2, Act represents the activation function. $W_{a+\text{1},l}$ denotes the weight of the l-th RG-Layer in the (a+1)-th RGAB block. The output features of the a-th RGAB block are combined with the output features of the 1, …, (l−1) RG-Layers in the (a+1)-th block to produce G feature maps. The structure, where the output of each layer of the previous RGAB and this block are all superimposed with those of all subsequent layers, enables the extraction of local dense features of the image [8] while reducing the network complexity.

Compared with the block using the concat operation to extract local dense features of the image, the RGAB reduces the number of output features of the l-th RG-Layer by a factor of (l−1) (when the input and output features of each RGAB block are equal), greatly reducing the model’s computational parameters and complexity (As mentioned in Section 2.1), thus achieving model lightweighting. Moreover, the output of each RG-Layer is passed in an additive manner to extract local dense features of the image.

The design of RGAB represents a paradigm shift from feature accumulation to feature refinement. In traditional concatenation-based dense blocks, the l−th layer has to process inputs with a channel number of C×l, leading to escalating computational demands. In contrast, our RGAB maintains a constant channel width. This allows the network to continuously enhance the most salient information within a fixed channel capacity, rather than simply expanding its representational space. This approach is inherently more parameter-efficient and computationally economical, directly addressing the core objectives of lightweight model design.

After inference through L RG-Layers in the (a+1)-th RGAB block, local feature fusion is needed. As shown in Fig 6, $F_{a}$ is the output features of the a-th RGAB block. $F_{a}$ and the outputs of its L RG-Layers in the (a+1)-th RGAB are concatenated through concat operation and then passed through a 1 × 1 convolution operation to control the output feature information.


$$F_{a+\text{1},LFF}={Conv}_{\text{1}\times \text{1}}\left[Concat\left(F_{a},F_{a+\text{1},\text{1}}\ldots \ldots ,F_{a+\text{1},L}\right)\right]$$ (3)

The number of features in $F_{a+\text{1},\;LFF}\;\text{and}\;F_{a}$ are the same. To further improve the information flow during network inference, the RGAB introduces local residual learning after local feature fusion. $F_{a+\text{1}}$, the output of the (a+1)-th RGAB block can be expressed as:


$$F_{a+\text{1}}=F_{a}+F_{a+\text{1},\;LFF}$$ (4)

### 2.3. R^2^GDN network structure

**Fig 7:**
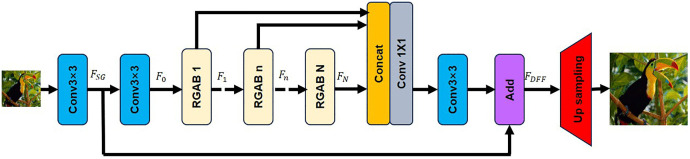
Architecture of the R^2^GDN network.

The R^2^GDN network is illustrated in Fig 7. The network is primarily composed of four components: the shallow feature extraction (SFE) block, repghost and addition based residual dense block (RGABs), the dense feature fusion (DFF), and the up-sampling block.

We denote the input image of the network as $I_{LR}$ and the output image as $I_{SR}$. Initially, shallow feature extraction is performed on the input image $I_{LR}$ using two convolutional layers. The first convolutional layer extracts feature $F_{SG}$ from $I_{LR}$.


$$F_{SG}={Conv}_{\text{3}\times \text{3}}\left(I_{LR}\right)$$ (5)

The features (Shallow Feature Extraction + Global Residual Learning) are used for further shallow feature extraction and global residual learning. The second convolutional layer takes $F_{SG}$ as input and further extracts shallow features. The output feature, denoted as $F_{\text{0}}$, is used as the input to the first RGAB.


$$F_{\text{0}}={Conv}_{\text{3}\times \text{3}}\left(F_{SG}\right)$$ (6)

Assuming the entire network has N RGAB blocks, we define the output features of the n-th RGAB block as $F_{n}$. $F_{n}$ can be expressed as:


$$\begin{array}{c} F_{n}=O_{RGAB,n}(F_{n-\text{1}})\\ F_{n-\text{1}}=O_{RGAB,n-\text{1}}(F_{n-\text{2}})\\ \begin{array}{c} \vdots \\ F_{\text{1}}=O_{RGAB,\text{1}}(F_{\text{0}}) \end{array} \end{array}$$ (7)

In formula 7, $O_{RGAB,n}$denotes the operation of the n-th RGAB. $O_{RAGB,n}$ is a composite function composed of depth-wise convolution, pointwise convolution, and a non-linear activation function. $F_{n}$ is obtained by the internal convolution operations within the n-th RGAB and is thus referred to as the local feature.

The dense feature fusion (DFF) is an operation that fuses the local features output by the n-th RGAB modules with the global residual feature $F_{SG}$. In the equation 8,$\;F_{DFF}$ represents the features output after the DFF operation, and $F_{Conv}$ denotes a series of 1 × 1 and 3 × 3 convolutional operations.


$$F_{DFF}=F_{Conv}\left(Concat\left(F_{\text{1}}\ldots \ldots F_{n}\right)\right)+F_{SG}$$ (8)

After obtaining the fused features in the low-resolution space, we perform the up sampling operation, which can be expressed as:


$$I_{SR}=Upsampling(F_{DFF})$$ (9)

We use sub-pixel convolution for upsampling. This technique overcomes the checkerboard effect problem associated with conventional upsampling methods (e.g., transposed convolution) by reordering the channels of low-resolution feature maps to generate high-resolution outputs. The fundamental concept is to create a feature map with a channel number equal to the square of the upsampling factor through convolution. The pixel shuffle operation subsequently reorganizes these channels to form the high-resolution output.

## 3. Experimental verification

### 3.1. Experimental settings

In this study, we used 800 high-quality RGB images from the DIV2K [32] dataset and 2000 high-quality RGB images from the Flickr2K [33] dataset as the training set. We evaluated the performance of our model on five benchmark datasets: Set5 [34] with 5 natural images representing different scenes and content; Set14 [35] containing 14 natural images with more diverse scenes and objects such as architecture, animals, and plants; BSD100 [36] with 100 high-resolution and high-quality natural scene images; Urban100 [37] with 100 urban landscape images featuring various buildings and streets; and Manga109 [38], a super-resolution dataset for manga images, including 109 high-quality manga images.

Low-resolution (LR) images were generated by down-sampling the high-resolution (HR) images using bicubic interpolation. The super-resolution results were evaluated using the Y-channel component of the images in the YCbCr color space, with metrics including Peak Signal-to-Noise Ratio (PSNR) and Structural Similarity Index (SSIM).

We set the size of the LR images to 64 × 64. For networks with different upscaling factors, the corresponding HR image patches were automatically cropped from the training images. During each training iteration, one HR image patch was cropped from each training image, and data augmentation was performed by randomly applying one of the following transformations: 90° rotation, horizontal flipping, or vertical flipping. The super-resolution network was implemented using the PyTorch framework and updated using the Adam optimizer. The learning rate for all layers was initialized to 10^−4^. After 750 training epochs, the learning rate was updated to 10^−5^; after 900 epochs, it was updated to 10^−6^; and the training was completed after 1000 epochs.

### 3.2. Network performance comparison

We compared our network with state-of-the-art methods: FSRCNN [17], VDSR [39], DRCN [18], EDSR-baseline [7], CARN [19], IMDN [20], RepRFN [21], SwinIR-light [9], RDN [8], CFIN [40], FIWHN [41], DMNet [42]. Specifically, the network using the structure shown in Fig 5(b) is denoted as R^2^GDN-GBU, while the network using the structure shown in Fig 5(c) is denoted as R^2^GDN. Table 1–2, and Table 3 present the quantitative comparisons of super-resolution reconstruction results for ×2, × 3, and ×4 upscaling factors, respectively. It can be observed that our R^2^GDN performs well on most datasets, particularly excelling in the Structural Similarity Index (SSIM) metric, surpassing the majority of the models.

**Table 1:** Quantitative comparison of different algorithms on five benchmark datasets with scale factor of 2.

Method	Scale	Params	Set5	Set14	BSD100	Urban100	Manga109
			PSNR/SSIM	PSNR/SSIM	PSNR/SSIM	PSNR/SSIM	PSNR/SSIM
FSRCNN [1 7]	×2	12K	37.00/0.9558	32.63/0.9088	31.53/0.8920	29.88/0.9020	36.67/0.9710
VDSR [39]		666K	37.53/0.9587	33.03/0.9124	31.90/0.8960	30.76/0.9140	37.22/0.9750
DRCN [18]		1,774K	37.63/0.9588	33.04/0.9118	31.85/0.8942	30.75/0.9133	37.55/0.9732
EDSR-baseline [7]		1,370K	37.99/0.9604	33.57/0.9175	32.16/0.8994	31.98/0.9272	38.54/0.9769
CARN [19]		1592K	37.76/0.9590	33.52/0.9166	32.09/0.8978	31.92/0.9256	38.36/0.9765
IMDN [20]		694K	38.00/0.9605	33.63/0.9177	32.19/0.8996	32.17/0.9283	38.88/0.9774
RepRFN [21]		386K	38.07/0.9612	33.63/0.9184	32.22/0.9009	32.10/0.9274	39.00/0.9779
SwinIR-light [9]		878K	38.14/0.9611	33.86/0.9206	32.31/0.9012	32.76/0.9340	39.12/0.9783
RDN [8]		22,000K	**38.24**/0.9614	**34.01**/0.9212	**32.34**/0.9017	**32.89**/**0.9353**	39.18/0.9780
CFIN [40]		675K	38.14/0.9613	33.80/0.9199	32.26/0.9006	32.48/0.9311	38.93/0.9347
FIWHN [41]		705K	38.16/0.9613	33.73/0.9194	32.27/0.9007	32.75/0.9337	39.07/0.9782
DMNet [42]		572K	38.23/0.9613	33.95/0.9209	32.31/0.9015	32.84/0.9347	**39.39**/**0.9786**
R^2^GDN-GBU (ours)		862K	37.60/0.9630	33.10/0.9209	31.86/0.9046	30.68/0.9183	38.24/0.9780
R^2^GDN (ours)		784K	37.74/**0.9640**	33.19/**0.9221**	31.93/**0.9058**	30.88/0.9204	38.38/0.9784

**Table 2:** Quantitative comparison of different algorithms on five benchmark datasets with scale factor of 3.

Method	Scale	Params	Set5	Set14	BSD100	Urban100	Manga109
			PSNR/SSIM	PSNR/SSIM	PSNR/SSIM	PSNR/SSIM	PSNR/SSIM
FSRCNN [1 7]	×3	12K	33.18/0.9140	29.37/0.8240	28.53/0.7910	26.43/0.8080	31.10/0.9210
VDSR [39]		666K	33.66/0.9213	29.77/0.8314	28.82/0.7976	27.14/0.8279	32.01/0.9340
DRCN [18]		1,774K	33.82/0.9226	29.76/0.8311	28.80/0.7963	27.15/0.8276	32.24/0.9343
EDSR-baseline [7]		1,555K	34.37/0.9270	30.28/0.8417	29.09/0.8052	28.15/0.8527	33.45/0.9439
CARN [19]		1,592K	34.29/0.9255	30.29/0.8407	29.06/0.8034	28.06/0.8493	33.50/0.9440
IMDN [20]		703K	34.36/0.9270	30.32/0.8417	29.09/0.8046	28.17/0.8519	33.61/0.9445
RepRFN [21]		392K	34.45/0.9280	30.39/0.8430	29.13/0.8068	28.06/0.8494	33.76/0.9451
SwinIR-light [9]		886K	34.62/0.9289	30.54/0.8463	29.20/0.8082	28.66/0.8624	33.98/0.9478
RDN [8]		22,000K	**34.71**/0.9296	**30.57**/0.8468	29.26/0.8093	**28.80**/0.8653	34.13/0.9484
CFIN [40]		681K	34.65/0.9289	30.45/0.8443	29.18/0.8071	28.49/0.8583	33.89/0.9464
FIWHN [41]		713K	34.50/0.9283	30.50/0.8451	29.19/0.8077	28.62/0.8607	33.97/0.9472
DMNet [42]		578K	**34.71**/0.9295	30.56/0.8459	29.26/0.8092	28.79/0.8640	**34.35**/**0.9488**
R^2^GDN-GBU (ours)		862K	33.75/0.9299	29.94/0.8495	29.37/0.8303	28.25/0.8629	32.75/0.9427
R^2^GDN (ours)		784K	33.92/**0.9319**	30.04/**0.8511**	**29.45/0.8319**	28.48/**0.8687**	33.07/0.9457

**Table 3:** Quantitative comparison of different algorithms on five benchmark datasets with scale factor of 4.

Method	Scale	Params	Set5	Set14	BSD100	Urban100	Manga109
			PSNR/SSIM	PSNR/SSIM	PSNR/SSIM	PSNR/SSIM	PSNR/SSIM
FSRCNN [1 7]	×4	12K	30.72/0.8660	27.61/0.7550	26.98/0.7150	24.62/0.7280	27.90/0.8610
VDSR [39]		666K	31.35/0.8838	28.01/0.7674	27.29/0.7251	25.18/0.7524	28.83/0.8870
DRCN [18]		1774K	31.53/0.8854	28.02/0.7670	27.23/0.7233	25.14/0.7510	28.93/0.8854
EDSR-baseline [7]		1518K	32.09/0.8938	28.58/0.7813	27.57/0.7357	26.04/0.7849	30.35/0.9067
CARN [19]		1592K	32.13/0.8937	28.60/0.7806	27.58/0.7349	26.07/0.7837	30.47/0.9084
IMDN [20]		715K	32.21/0.8948	28.58/0.7811	27.56/0.7353	26.04/0.7838	30.45/0.9075
RepRFN [21]		402K	32.28/0.8969	28.68/0.7836	27.65/0.7389	26.18/0.7858	30.79/0.9102
SwinIR-light [9]		897K	32.44/0.8976	28.77/0.7858	27.69/0.7406	26.47/0.7980	30.92/**0.9151**
RDN [8]		22000K	32.47/**0.8990**	28.81/0.7871	27.72/0.7419	**26.61**/**0.8028**	31.00/**0.9151**
CFIN [40]		699K	32.49/0.8985	28.74/0.7849	27.68/0.7396	26.39/0.7946	30.73/0.9124
FIWHN [41]		725K	32.30/0.8967	28.76/0.7849	27.68/0.7400	26.57/0.7989	30.93/0.9131
DMNet [42]		587K	**32.51**/0.8987	**28.84**/0.7866	**27.73**/0.7410	26.58/0.7991	**31.14**/0.9150
R^2^GDN-GBU (ours)		862K	31.52/0.8948	28.22/0.7889	27.32/0.7454	25.26/0.7698	29.80/0.9039
R^2^GDN (ours)		784K	31.61/0.8960	28.25/**0.7894**	27.35/**0.7461**	25.32/0.7718	29.87/0.9049

The Structural Similarity Index (SSIM) measures the similarity between images based on three relatively independent metrics: luminance, contrast, and structure. The superior performance of our model in this metric indicates its ability to better restore structural information in images, such as edges and textures, thereby providing a more natural and visually pleasing result that aligns better with human perception.

As shown in Table 1, R²GDN achieves better objective evaluation metrics in the 5 benchmark test sets for the × 2 reconstruction task. Compared with performance-oriented models (such as RDN), the number of model parameters of R²GDN is reduced by 96.4%, but the SSIM of R²GDN (taking BSD100 as an example) is 0.9058, which is higher than that of RDN (0.9017). Compared with other lightweight models (such as CARN, IMDN, RepRFN), the structural similarity index of R²GDN (taking BSD100 as an example) is increased by 0.89%, 0.69%, and 0.54%, respectively.

As shown in Table 2, compared with performance-oriented models (such as RDN), the SSIM of R²GDN (taking BSD100 as an example) is 0.8319, which is higher than that of RDN (0.8093). Compared with other lightweight models (such as CARN, IMDN, RepRFN), the structural similarity index of R²GDN (taking BSD100 as an example) is increased by 3.55%, 3.41%, and 3.11%, respectively.

As shown in Table 3, compared with performance-oriented models (such as RDN), the SSIM of R²GDN (taking BSD100 as an example) is 0.7461, which is higher than that of RDN (0.7419). Compared with other lightweight models (such as CARN, IMDN, RepRFN), the structural similarity index of R²GDN (taking BSD100 as an example) is increased by 1.53%, 1.47%, and 0.97%, respectively.

On the basis of achieving good super-resolution reconstruction results, our model has found a better balance between performance and lightweight design. Compared with high-performance super-resolution networks, our R^2^GDN can achieve comparable PSNR and SSIM values with fewer parameters and even outperforms some of these state-of-the-art models in certain metrics. Compared with advanced lightweight networks, R^2^GDN not only has a small number of parameters but also achieves better evaluation metrics on some datasets.

Compared to GhostSR [28], which directly employs the original Ghost module, which maintains a consistent structure during both training and inference. This structure includes regular convolutions to generate “essential features” and inexpensive shift operations to create “ghost features,” which are then merged via concatenation. The fundamental unit structure of R²GDN (RG-Layer) innovatively integrates structural reparameterization with the Ghost concept. During training, the RG-Layer boasts a richer set of branches and stronger expressive power; during inference, it consolidates into a single standard convolution. When stacking block structures, we opt for the more efficient addition operation over concatenation, reducing latency and promoting feature refinement.

Compared to RepRFN [21], which explicitly fuses multi-scale features through a complex multi-branch structure, offering strong performance but at the cost of increased structural complexity. In contrast, R²GDN adopts a “refinement” mode, repeatedly enhancing features within a fixed channel dimension through additive dense connections, thereby achieving greater simplicity and efficiency. Unlike the concatenation operation used by RepRFN (and most traditional dense networks), we employ addition for feature fusion within the RGAB module. This fundamentally prevents channel expansion, significantly reducing computational complexity and memory access overhead. Experimental results indicate that, despite having slightly more parameters than RepRFN, R²GDN achieves a notable improvement in structural similarity (for example, in ×4 super-resolution on BSD100, the SSIM of R²GDN is 0.7461 compared to 0.7389 of RepRFN).

**Fig 8:**
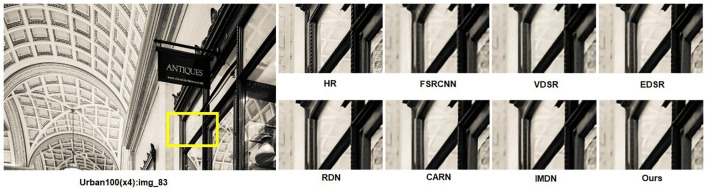
Reconstruction results comparison of typical algorithms and R^2^GDN on Urban100(083) dataset with scale factor of 4.

**Fig 9:**
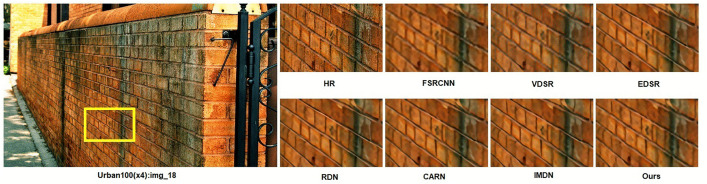
Reconstruction results comparison of typical algorithms and R^2^GDN on Urban100(018) dataset with scale factor of 4.

Fig 8 and Fig 9 presents ×4 visual comparisons on the Urban100 dataset. For the images “img_18” and “img_83” from the Urban100 dataset, it can be observed that the grid structures are better restored compared to other methods. This also demonstrates the effectiveness of our R^2^GDN.

We selected typical networks for a comparative experiment on partial image reconstruction results at ×4 super-resolution. As shown in the images “img_18” and “img_83” from the Urban100 dataset, the subjective visual effects reveal that our method can achieve results comparable to or even better than state-of-the-art methods such as RDN, as well as lightweight methods like FSRCNN, VDSR, EDSR-baseline, CARN, and IMDN. Our approach effectively restores image edges and textures, making details clear and visible. Particularly for the reconstruction of “img_83,” our network accurately reconstructs the textures of the glass reflections. This visual comparison further illustrates that the super-resolution results of our proposed R^2^GDN have reached an advanced level and can meet the requirements of practical applications.

**Fig 10:**
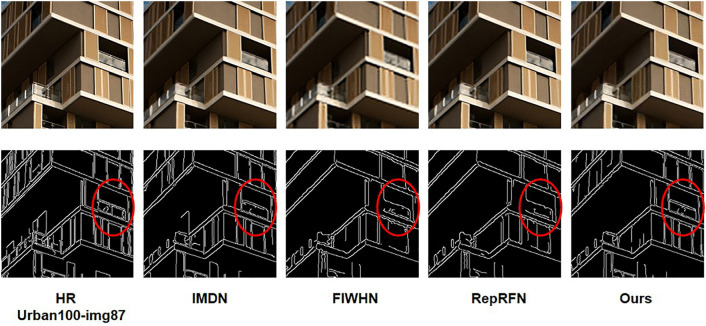
Edge extraction on the reconstructed image of typical algorithms and R²GDN on Urban100 (img_87) with scale factor of 4.

As shown in Tables 1–3 to 3, R²GDN achieves higher SSIM compared to other models, but has relatively lower PSNR indices. Through an analysis of the definitions of SSIM and PSNR, as well as an in-depth study of the reconstructed images, we have determined that the image reconstruction performance of R²GDN is more inclined towards enhancing the structural information of images rather than pursuing absolute pixel value matching. Studies have already confirmed that in the task of image super-resolution reconstruction, a higher PSNR does not necessarily correspond to better image reconstruction quality. Some reconstructed images have high PSNR values, but their details are overly smooth, leading to a worse intuitive perception. We performed edge extraction on the reconstructed images, and the results are shown in Fig 10. The results indicate that the reconstructed images of R²GDN have richer edge information compared to other models. At the pixel level, these restored details may not be entirely consistent with the original image, thereby increasing the mean squared error (MSE) and decreasing the PSNR.

### 3.3. Ablation Investigation

We designed a series of ablation experiments to analyze the effectiveness of each module in the model. The network was trained using images of size 64 × 64 and updated with the Adam optimizer, with an initial learning rate of 10^−4^. After 750 training epochs, the learning rate was updated to 10^−5^; after 900 epochs, it was updated to 10^−6^, and the training was completed after 1000 epochs. We evaluated the × 4 super-resolution reconstruction performance on three benchmark datasets: Set14, BSD100, and Urban100. The results of the ablation experiments for R^2^GDN are summarized in Table 4.

**Table 4:** Comparison of model parameters and performance for different numbers of RGAB in R^2^GDN.

RGAB	Params	Set14	BSD100	Urban100
		PSNR/SSIM	PSNR/SSIM	PSNR/SSIM
8	647K	28.18/0.7878	27.31/0.7448	25.22/0.7680
12	784K	**28.25/0.7894**	**27.35/0.7461**	**25.32/0.7718**
16	921K	28.22/0.7888	27.33/0.7455	25.26/0.7697
32	1469K	27.56/0.7733	26.93/0.7325	24.43/0.7367

To further validate the contribution of redundant features generated by different low-cost operations to model performance and to demonstrate the advancement of the structure designed in this paper, we conducted replacement tests on low-cost operations in the lightweight layer based on the R^2^GDN model. We compared the results with identity mapping, batch normalization, GBU, and 1 × 1 convolution. The experimental results show that the lightweight layer structure using 1 × 1 convolution, as adopted in this paper, achieves better results in both objective metrics and subjective evaluation, with the metrics statistics presented in the Table 5.

**Table 5:** Comparison of objective indicators of reconstruction effect when using different low-cost operations.

Identity	BN	GBU	Conv 1 × 1	Set5	Set14	BSD100	Urban100
				PSNR/SSIM	PSNR/SSIM	PSNR/SSIM	PSNR/SSIM
√	×	×	×	31.03/0.8851	27.92/0.7811	26.59/0.7243	24.89/0.6686
×	√	×	×	30.24/0.8686	27.35/0.7672	26.05/0.6653	26.70/0.7612
×	×	√	×	31.52/0.8948	28.22/0.7889	27.32/0.7454	25.26/0.7698
×	×	×	√	31.61/0.8960	28.25/0.7894	27.35/0.7461	25.32/0.7718

When the model structure is kept the same, this paper adopts 1x1 convolution as a low-cost operation. Compared to the use of BN structure, the average enhancement in objective evaluation metrics is 3.4%; compared to identity mapping, the average enhancement in objective evaluation metrics is 3.56%; compared to GBU, the average enhancement in objective evaluation metrics is 0.17%. Ablation experiments have shown that the model designed in this paper has certain structural innovation and performance benefits in the task of image super-resolution reconstruction.

Through our in-depth ablation studies, we discovered that the complexity of the Gated BN Unit (GBU) does not correspond to the performance improvements it offers. Consequently, we conducted a more comprehensive optimization: we eliminated the BN and the entire GBU structure and determined that employing a parallel 1 × 1 convolution branch as a low-cost operation achieves superior outcomes. The 1 × 1 convolution is a lightweight yet parameter-rich operation that not only offers linear transformation capabilities beyond identity mapping, aiding in gradient flow improvement, but also integrates seamlessly and cleanly into our reparameterization framework—during inference, it can be seamlessly merged with the main branch’s 3 × 3 convolution into a single, standard convolutional layer, without leaving any additional, non-standard operations.

As shown in Tables 1–3 to 3, sometimes a small average difference in the SSIM metric alone is not sufficient to conclusively demonstrate the superiority of a model. We conducted paired sample t-tests for the reconstruction objective metric SSIM of R^2^GDN, RepRFN, FIWHN, CFIN, and SwinIR-light on the BSD100 test set under the × 4 scale. We also calculated the 95% confidence intervals for all key comparisons to assess the statistical significance of these differences. The results are shown in Table 6.

**Table 6:** Statistics comparison of SSIM between R2GDN and typical algorithms on BSD100 (×4).

Model Comparison	Paired Differences				Significance
	Mean	Standard Deviation	95% Confidence Interval of the Difference		Two-Tailed p-value
			Lower Bound	Upper Bound	
R^2^GDN—CFIN	0.00959	0.17081	-0.02431	0.43480	0.576
R^2^GDN—FIWHN	0.4434	0.17390	0.00983	0.78840	0.012
R^2^GDN—RepRFN	0.1698	0.85330	0.00005	0.03391	0.049
R^2^GDN—SwinIR-light	0.5316	0.22641	0.00824	0.9809	0.021

Compared with RepRFN, R^2^GDN has an average SSIM difference of +0.0170, with a 95% confidence interval of [0.00005, 0.03391] and a two-sided p-value of 0.049. Statistics indicate that the difference in the reconstruction objective metric SSIM between R^2^GDN and RepRFN reaches the level of statistical significance (p < 0.05). More importantly, the lower limit of its 95% confidence interval is greater than zero, which provides us with 95% confidence that the performance advantage of R^2^GDN is real. Although this advantage may seem small numerically, statistical tests confirm its systematic nature rather than randomness.

Compared with FIWHN, R^2^GDN has an average SSIM difference of +0.0443, with a 95% confidence interval of [0.0098, 0.0788] and a two-sided p-value of 0.012. Statistics show that the difference in the reconstruction objective metric SSIM between R^2^GDN and FIWHN reaches the level of statistical significance (p < 0.05) and is significant. Compared with SwinIR-light, R2GDN has an average SSIM difference of +0.0532, with a 95% confidence interval of [0.0082, 0.0981] and a two-sided p-value of 0.021. Statistics indicate that the difference in the reconstruction objective metric SSIM between R^2^GDN and SwinIR-light reaches the level of statistical significance (p < 0.05) and is significant.

Compared with CFIN, R^2^GDN has an average SSIM difference of +0.0096, with a 95% confidence interval of [−0.0243, 0.0435] and a two-sided p-value of 0.576. Statistics show that the difference in the reconstruction objective metric SSIM between R^2^GDN and CFIN does not reach the level of statistical significance (p > 0.05). Statistical conclusions indicate that there is no significant difference in performance between R^2^GDN and CFIN.

In summary, in response to the questions you raised, the statistical evidence we provided strongly supports the conclusion that “our R^2^GDN model significantly outperforms RepRFN, FIWHN, and SwinIR in terms of the SSIM metric.”

### 3.4. Inference time

**Fig 11:**
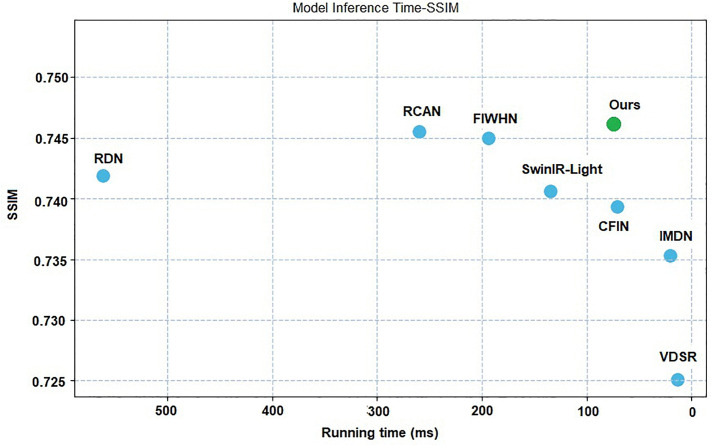
Average running time and structural similarity index on the BSD100 dataset (×4) using a high – performance GPU device.

The inference time is an extremely important metric for lightweight image super-resolution reconstruction algorithms. To further validate that the proposed R^2^GDN network is lightweight, we conducted comparative experiments on reconstruction speed using both high-performance GPU devices and edge devices. The experiments were performed on the BSD100 dataset (×4) with test images of size 64 × 64 pixels. We first performed a model warm-up operation, as the initial inference time may include network loading time. Subsequently, we ran the model 10 times to measure the average inference time. The results are shown in Fig 11, Tables 7 and 8.

**Table 7:** Average inference time on BSD100(×4) using high-performance GPU.

Method	Params	SSIM	Average running time
RCAN [43]	15592K	0.7455	260.2ms
RDN [8]	22000K	0.7419	561.4ms
SwinIR-light [9]	897K	0.7406	134.9ms
CFIN [40]	699K	0.7396	**65.5ms**
FIWHN [4 1]	725K	0.7400	197ms
R^2^GDN	784K	**0.7461**	73.9ms

**Table 8:** Average inference time on BSD100(×4) using edge devices.

Method	Params	SSIM	Average running time
RDN [8]	22000K	0.7419	636.7ms
IMDN [20]	715K	0.7353	**65.4ms**
SwinIR-light [9]	897K	0.7406	179.3ms
CFIN [40]	699K	0.7396	105.7ms
FIWHN [4 1]	725K	0.7400	307.6ms
R^2^GDN	784K	**0.7461**	91.1ms

High-performance GPU inference test was profiled on an NVIDIA GeForce RTX 4090. Edge-hardware testing was performed on the Jetson Nano B01 developer kit (NVIDIA Jetson family), integrating a quad-core ARM Cortex-A57 MP Core application processor and a 128-core NVIDIA Maxwell graphics processing unit.

### 3.5. Computational complexity

The computational complexity of a neural network model refers to the computational resources and time required for the model to operate, which typically includes both time complexity and space complexity. Time complexity primarily measures the speed of model operation, the amount of computation required to complete one forward pass or training iteration, common metrics include: FLOPs (Floating Point Operations). Space complexity measures the memory or storage resources required for the model; common metrics include: number of parameters, as shown in Tables 1–3 to 3. FLOPs refer to the number of floating-point operations, which is a key indicator of computational complexity. It represents the total number of floating-point additions, multiplications, and other operations required during computation. Table 9 presents the FLOPs of the R^2^GDN and other advanced models at a magnification factor of 2. The comparison shows that R^2^GDN can achieve low computational complexity while ensuring stable image reconstruction performance.

**Table 9:** Comparison of FLOPs between R^2^GDN and other advanced methods during 2 × super-resolution reconstruction (FLOPs is measured on up-sampled images with a spatial size of 1280 × 720 pixels. SSIM is tested on BSD100).

Method	FLOPs	SSIM
CARN [19]	222.8G	0.8978
IMDN [20]	158.8G	0.8996
DMNet [42]	**115.7G**	0.9015
SwinIR-light [9]	195.6G	0.9012
CFIN [40]	116.9G	0.9006
FIWHN [4 1]	137.7G	0.9007
R^2^GDN (ours)	146.9G	**0.9058**

### 3.6. Evaluation of datasets in different fields

In order to validate the generalizability of R^2^GDN, we performed super-resolution reconstruction network training and image reconstruction experiments using IC microscopic images. The training set is composed of REFICS [13] and a portion of self-collected images. REFICS is a large-scale synthetic scanning electron microscope (SEM) dataset, which includes 800,000 SEM images spanning 32nm and 90nm node technologies. We chose 5,000 images with minimal noise and high clarity from the active area, polysilicon, and metal layers in REFICS, and combined them with 3,000 self-collected high-definition integrated circuit microscopic images to form the training set. Self-collected high-definition micrographs of IC were acquired from two distinct devices. The first device is fabricated in a 0.18 µm 1P6M Bipolar-CMOS-DMOS (BCD) process; its images were captured at 1,800 × magnification using an optical electron microscope. The second device is manufactured in a 55 nm 1P5M Bipolar-CMOS technology; its images were obtained at 200,000 × magnification via scanning electron microscopy.

**Fig 12:**
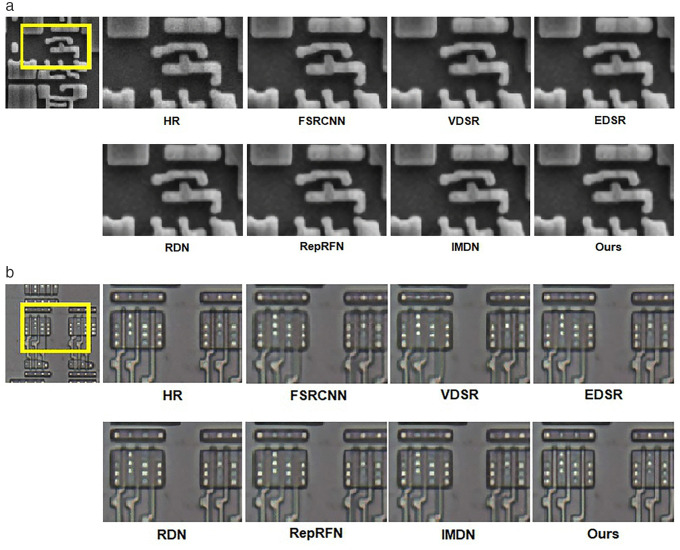
Reconstruction results comparison of typical algorithms and R²GDN on IC dataset with ×4 scale.

We utilized 80 self-collected images that do not overlap with the training set as the overall test set, 50 metal layer images, 50 poly layer images, and 50 diffusion area (DF) images that do not overlap with the training set form the independent test sets. We retrained several typical networks on our IC training dataset for reconstruction performance comparison. The objective performance indicators of our model on ×4 scale are com-pared with other typical model indicators as shown in Table 10. The visual effects of super-resolution reconstruction of IC microscopic images by R^2^GDN are demonstrated in Fig 12.

**Table 10:** Quantitative comparison of different algorithms on IC test sets with scale factor of 4.

Model	Scale	Overall	Metal	DF	Poly
		SSIM/LPIPS	SSIM/LPIPS	SSIM/LPIPS	SSIM/LPIPS
FSRCNN	×4	0.9358/0.2738	0.9630/0.2430	0.8870/0.3370	0.8930/0.1576
CARN		0.9523/0.1331	0.9763/0.1182	0.9411/0.1961	0.9167/0.0142
EDSR-baseline		0.9496/0.1871	0.9281/0.1173	0.8943/0.1907	0.9156/0.0131
IMDN		0.9471/0.1332	0.9712/0.1197	0.9488/0.1956	0.9131/0.0154
RDN		0.9478/0.1306	0.9757/**0.1133**	0.9620/0.1909	**0.9243**/**0.0127**
R^2^GDN (ours)		**0.9556**/**0.1236**	**0.9771**/0.1168	**0.9637**/**0.1869**	0.9239/0.0129

As indicated in Table 10, in the × 4 reconstruction task for IC circuit microscopic images, R²GDN attains the highest SSIM index in the overall test set, metal test set, and DF test set, and the second-highest SSIM index in the poly test set, compared with other top-performing models. We also performed a perceptual evaluation comparison of the IC reconstruction results. R²GDN achieves the lowest LPIPS (Learned Perceptual Image Patch Similarity) value in the overall test set and active region test set, and the second-lowest LPIPS value in the metal test set and poly test set. The results show that R²GDN has achieved outstanding performance in both structural fidelity (highest SSIM) and visual perceptual quality (lowest LPIPS).

The primary focus for engineers inspecting the microscopic structure of integrated circuits is on the structural characteristics of the circuit, such as linewidths and edges within the circuit. The structural similarity measured by SSIM directly meets these requirements. A high SSIM index indicates that the reconstructed IC microscopic images are more consistent with the actual situation of key structural information, including line shapes and edge locations. If a model that solely aims to achieve high PSNR results in overly smooth edges in the microscopic images, integrating such a model into the IC microscopic image acquisition process would be counterproductive.

## 4. Conclusions

Aiming to address the issues of high computational complexity and substantial memory consumption in existing super-resolution reconstruction networks, this paper proposes a lightweight image super-resolution network based on feature reuse and structural reparameterization techniques. This approach makes super-resolution networks more suitable for deployment on edge devices, enabling fast and accurate extraction of local-global deep features from images. Specifically, we leverage intrinsic features to generate redundant features via inexpensive operations and employ structural reparameterization techniques to design a feature-reusing reparametrized layer, termed the RG-Layer. Building on this, we design an efficient deep feature extraction module, the Residual Gated Block (RGAB), which maintains dense connections, local feature fusion, and local residual learning while using an Addition feature fusion method. These components are integrated into a highly efficient image super-resolution network, the Reparametrized Gated Dense Network (R^2^GDN). Experimental results demonstrate that R^2^GDN achieves a good balance between performance and network complexity compared to other state-of-the-art algorithms. However, a comparison of the reconstruction results reveals that the proposed model still exhibits some issues, such as blurred edges and artifacts in the reconstructed images. In the future, we will focus on improving the image quality of the reconstructed results while further ensuring the network’s lightweight nature to better fit edge devices.
